# Successful treatment of metastatic urothelial carcinoma arising in a transplanted renal allograft with paclitaxel, cisplatin, and gemcitabine combination therapy: a case report

**DOI:** 10.1186/s13104-015-0982-6

**Published:** 2015-02-04

**Authors:** Yasuyuki Kojima, Yuko Takahi, Naotsugu Ichimaru, Masayoshi Okumi, Shiro Takahara, Norio Nonomura

**Affiliations:** Department of Urology, Inoue Hospital, 16-17 Enoki-Cho, Suita, 564-0053 Osaka Japan; Department of Advanced Technology for Transplantation, Osaka University Graduate School of Medicine, Suita, Osaka Japan; Department of Urology, Osaka University Graduate School of Medicine, Suita, Osaka Japan

**Keywords:** Urothelial carcinoma, Paclitaxel, Cisplatin, Gemcitabine, Kidney transplantation, Hemodialysis

## Abstract

**Background:**

For locally advanced or metastatic urothelial carcinoma, cisplatin-based chemotherapy is the standard regimen. Nevertheless, almost all responding patients experience recurrence within the first year. When patients who have received prior cisplatin-based therapy become resistant, combination therapy with gemcitabine and paclitaxel has been reported. Few published case reports have addressed the utility of paclitaxel/cisplatin/gemcitabine combination therapy as second-line chemotherapy for advanced or metastatic urothelial carcinoma. This is the first report describing paclitaxel/cisplatin/gemcitabine combination therapy for metastatic urothelial carcinoma arising in a transplanted renal allograft and leading to a successful outcome.

**Case presentation:**

We present a case of metastatic urothelial carcinoma of a renal allograft in a 32-year-old Japanese man with a history of kidney transplantation ten years prior. Because the patient’s serum creatinine increased, hemodialysis was resumed, and the surgical allograft was removed. Multiple lung metastases were resistant to gemcitabine/cisplatin adjuvant chemotherapy, so paclitaxel/cisplatin/gemcitabine combination chemotherapy was instituted. After paclitaxel/cisplatin/gemcitabine chemotherapy, all pulmonary metastatic tumors disappeared. The patient has survived without disease progression for more than four years since treatment.

**Conclusion:**

Paclitaxel/cisplatin/gemcitabine combination therapy may be effective and lead to a survival advantage in patients with locally advanced or metastatic urothelial carcinoma when used as second-line chemotherapy following cisplatin-based therapy. However, further investigations may be required to confirm and evaluate the significance of this treatment.

**Electronic supplementary material:**

The online version of this article (doi:10.1186/s13104-015-0982-6) contains supplementary material, which is available to authorized users.

## Background

For locally advanced or metastatic urothelial carcinoma (UC), methotrexate/vinblastine/doxorubicin/cisplatin (MVAC) [1] or gemcitabine/cisplatin (GC) [2] combination chemotherapy represents the standard regimen. However when patients who have received prior cisplatin-based therapy become resistant, there is no standard regimen for second-line chemotherapy.

In general, malignant tumors occur more frequently in renal transplant recipients receiving immunosuppressive therapy. These malignancies are often more aggressive and are associated with a poor prognosis [3-5].

We report a case of metastatic UC of a renal allograft in a patient who showed long-term recurrence-free survival after being treated with a combination of paclitaxel, cisplatin, and gemcitabine (PCG).

## Case presentation

A 32-year-old Japanese man presented with gross hematuria. He had undergone a kidney transplant ten years earlier for end-stage kidney disease with focal segmental glomerulosclerosis at that time, he received a living kidney allograft from his 51-year-old father. On presentation, an abdominal computed tomography (CT) scan revealed a mass of approximately 4 cm in the allograft collecting system. Chest CT showed multiple lung metastases. The results of annual screening with abdominal CT and ultrasonography had previously been normal. The patient’s Eastern Cooperative Oncology Group (ECOG) performance status was 1 at this time.

Ureteroscopic biopsy was performed, and histopathology showed a grade 3, urothelial carcinoma. The clinical diagnosis was renal pelvis cancer of stage T2N0M1 according to the TNM classification of Malignant Tumors (7th edition).

After hemodialysis was resumed, we planned GC neoadjuvant chemotherapy. Gemcitabine (GEM) was administered at 1,000 mg/m^2^ on non-dialysis days 1, 8, and 15. Cisplatin (CDDP) was given intravenously at 35 mg/m^2^ on dialysis day 2. This regimen was repeated every four weeks.

**Figure 1:**
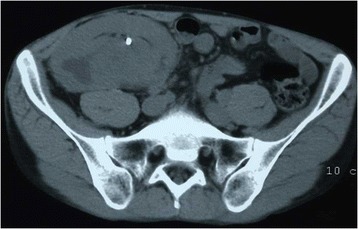
**Abdominal computed tomography (CT) scan of the patient.** Abdominal CT scan prior to surgery showed a mass measuring 6.4 × 3.7 cm in the transplanted kidney. A double-J ureteral stent was placed.

**Figure 2:**
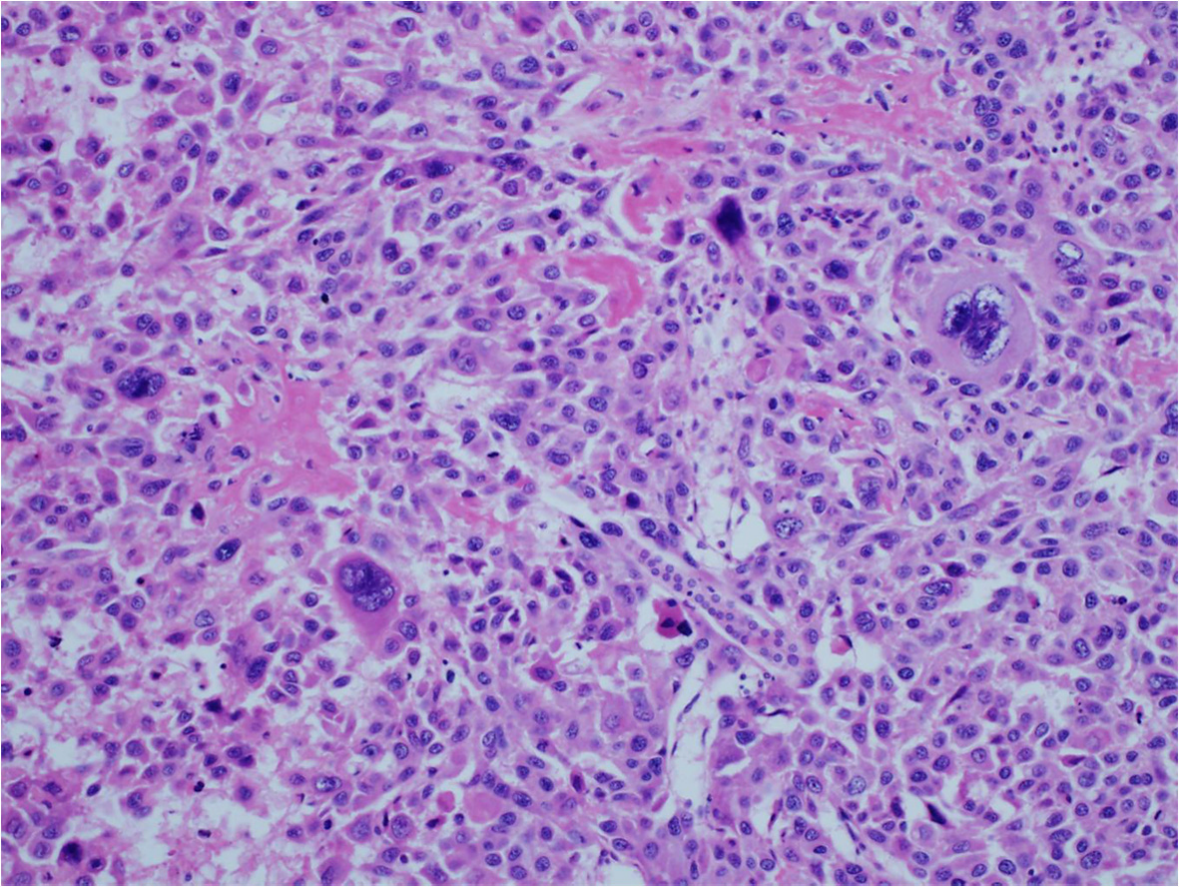
**Hematoxylin & eosin (HE) staining.** Microscopic appearance showed urothelial carcinoma, grade3. Some of the nuclei were very large, irregular, and hyperchromatic.

However, the tumor showed progression following two courses of GC treatment (Figure 1), so we decided to perform renal allograftectomy. Histopathological examination confirmed a grade 3 invasive UC with final staging of pT3 (Figure 2).

After the surgery, one course of postoperative GC treatment was administered. Evaluation with chest CT according to RECIST guidelines (version 1.1) showed increased size of the pulmonary metastatic tumors, judged as progressive disease (PD). As a result, paclitaxel/cisplatin/gemcitabine (PCG) combination therapy was started. The PCG protocol consisted of GEM 1,000 mg/m^2^ and paclitaxel (PTX) 80 mg/m^2^ on non-dialysis days 1 and 8 plus CDDP 35 mg/m^2^ on dialysis day 2. This course was repeated every three weeks.

**Figure 3:**
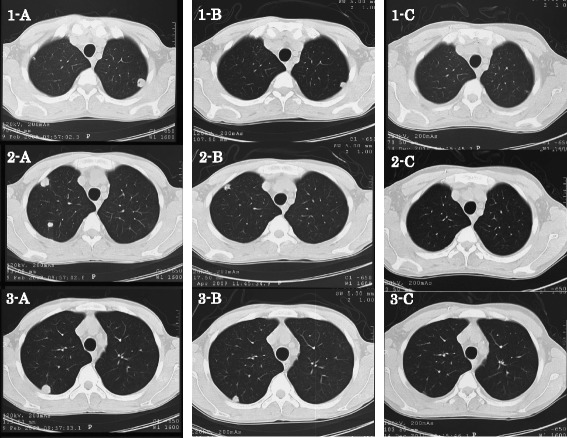
**Three phases of chest CT scans showing the metastatic tumors. A**, Before paclitaxel, cisplatin, and gemcitabine (PCG) chemotherapy. **B**, Reduction of the tumors after two courses of PCG. **C**, Disappearance of all metastatic tumors after seven courses of PCG.

After two courses of PCG, pulmonary metastatic tumors showed a 45% size reduction on chest CT, judged as a partial response (PR). After seven courses of PCG chemotherapy, CT demonstrated the disappearance of all pulmonary metastatic tumors, judged as a complete response (CR) (Figure 3). Toxicity was evaluated using the Common Terminology Criteria for Adverse Events (CTCAE) as a grade 2 neutropenia. There was no severe toxicity. Currently, the patient has been tumor-free for 54 months following treatment.

## Discussion

The overall incidence of de novo malignancies after renal transplantation is much higher than that in the general population because the former patients receive immunosuppressive therapy [6]. The risk of post-transplant UC is higher among patients with aristolochic acid nephropathy (AAN) (also called Chinese-herb nephropathy) [7] and those among patients with analgesic nephropathy (AN) because of prior analgesic abuse [8]. Several cases of UC (bladder, ureter, and renal pelvis involvement) have been reported in renal transplant patients without AAN or AN [9,10]. Renal pelvis cancer following a kidney transplantation almost occurs in the native kidney. The cause is unknown, but post-transplant UC of a renal allograft rarely occurs in the long-term postoperative period [11-13]. To detect malignancies, an intensive follow-up regimen including urine cytology should be adopted after renal transplantation [14].

MVAC combination chemotherapy is the standard regimen for locally advanced or metastatic UC [1]. A combination of gemcitabine and cisplatin (GC) has shown similar survival times compared with MVAC but with better safety [2]. GC is now considered to be another standard regimen for patients with locally advanced or metastatic UC.

However, an effective treatment strategy has not yet been established for patients who show resistance to initial treatment such as GC therapy. Some authors have reported the efficacy of gemcitabine/paclitaxel (GEM/PTX) combination therapy and the PCG combination therapy that we evaluated in our study [15-17].

The overall response rate (complete or partial) with GEM/PTX treatment following initial cisplatin or carboplatin-based therapy ranges from 60 to 66.7% [15,16]. With PCG therapy, an overall response rate of 77.6% and median survival time of 24 months have been reported, and 8.6% of the patients in this study had received prior adjuvant chemotherapy [17]. A randomized phase III study has been performed looking at the use of GC and PCG for locally advanced or metastatic UC; the PCG response rate was 55.5%, significantly higher than the 43.6% response rate with GC therapy [18]. In this study, the addition of paclitaxel to the GC combination provided a 3.1-month survival benefit (15.8 months median overall survival for PCG versus 12.7 months for GC), but this did not reach significance. Our case report shows that patients can survive for a long-period of time after PCG treatment. Thus, PCG has potential as a novel first or second-line chemotherapy regimen for locally advanced or metastatic UC.

It is important to be able to predict which regimen is effective in patients with UC before the initiation of chemotherapy, and we should be able to avoid ineffective treatment. The survival rate of metastatic bladder cancer patients treated with either GC or PCG is significantly higher among patients with low ERCC1 levels [19]. ERCC1 has been reported to be an independent prognosis prediction factor connected with platinum-based therapy for UC [19]. RRM1 has also been identified as a significant indicator of survival in patients receiving GC, with low expression levels correlating with greater survival benefit [20]. However, biomarkers indicating which regimen, GC or PCG, was more effective were not expressed.

In this way, personalized medicine based on biomarkers has not been completely established yet, it would make a remarkable contribution to cancer therapy in the near future.

For dialysis patients, although it is possible to give GEM and PTX on non-hemodialysis days at the same dosage as in patients with normal renal function, it is necessary to reduce the dose of cisplatin by 50% to avoid nephrotoxicity; thus, hemodialysis initiation one hour after injection is recommended [21,22]. With these modifications, the plasma level of free cisplatin available for anti-tumor activity is almost the same as in patients with normal renal function [22]. There have been no reports of PCG treatment for hemodialysis patients; however, PCG was safely performed in our case.

## Conclusions

The prognosis for metastatic UC is poor, with an average survival of less than six months for untreated patients [23]. Therefore, effective chemotherapy is needed for advanced or metastatic UC. This is the first report describing PCG combination therapy for metastatic UC arising in a transplanted renal allograft. PCG may have shown successful outcomes in our reported case, but further analyses on larger patient groups would be required to provide stronger evidence to support this therapeutic intervention.

## Consent

Written informed consent was obtained from the patient for publication of this Case Report and any accompanying images. A copy of the written consent is available for review by the Editor-in-Chief of this journal.

## Abbreviations

UC: Urothelial carcinoma
MVAC: Methotrexate/vinblastine/doxorubicin/cisplatin
GC: Gemcitabine/cisplatin
PCG: Paclitaxel/cisplatin/gemcitabine
PTX: Paclitaxel
CDDP: Cisplatin
GEM: Gemcitabine
